# Prevalence and outcomes of extrahepatic primary malignancy associated with Hepatocellular Carcinoma in a Korean population

**DOI:** 10.1186/s12885-015-1169-1

**Published:** 2015-03-18

**Authors:** Sukho Hong, Sook-Hyang Jeong, Sang Soo Lee, Jung Wha Chung, Sung Wook Yang, Seong Min Chung, Eun Sun Jang, Jin-Wook Kim, Jee Hyun Kim, Haeryoung Kim, Jai Young Cho, Yoo-Seok Yoon, Ho-Seong Han

**Affiliations:** 1 Department of Internal Medicine, Seoul National University College of Medicine, Seoul National University Bundang Hospital, 82, Gumi-ro 173 beon-gil, Bundang-gu, Seongnam-si, Gyeonggi-do, Republic of Korea; 2 Pathology, Seoul National University Bundang Hospital, Seoul National University College of Medicine, Gyeonggi-do, Republic of Korea; 3 Surgery, Seoul National University Bundang Hospital, Seoul National University College of Medicine, Gyeonggi-do, Republic of Korea

**Keywords:** Hepatocellular carcinoma, Multiple primary neoplasms, Mortality, Korea

## Abstract

**Background:**

With advances in hepatocellular carcinoma (HCC) screening and treatment, the incidence of diagnosing a case of extrahepatic primary malignancy (EHPM) in patients with HCC has increased. This study aimed to elucidate the prevalence and clinical outcomes of EHPM in patients with HCC who underwent curative resection in Korea.

**Methods:**

The clinical data of 250 patients with HCC who underwent curative resection in our hospital from May 2003 to December 2011 were retrospectively analyzed. The clinical features, overall survival, and causes of death were compared between patients with HCC with or without EHPM.

**Results:**

The prevalence of EHPM among the 250 patients was 13.2% (n = 33). The most common site of EHPM was the colorectal (n = 10), followed by the stomach (n = 9), breasts (n = 4), and kidneys (n = 3). Patients with EHPM were significantly older, and they presented with higher rates of comorbidities, a different etiology of HCC, and better liver function than patients without EHPM. Interestingly, overall survival was significantly lower in the EHPM group, which more frequently displayed extrahepatic causes of death. Moreover, the presence of EHPM was an independent factor for overall survival in the study population.

**Conclusions:**

The prevalence of EHPM in patients with HCC who underwent curative surgical resection was 13.2% in Korea, with colorectal and stomach cancers comprising most EHPMs (88%). The patients with EHPM displayed significantly worse survival because of extrahepatic causes of death, which should be considered in the management of HCC in the future.

## Background

Hepatocellular carcinoma (HCC) is the fifth common cancer globally and the third leading cause of cancer mortality [1]. Previously, extrahepatic primary malignancy (EHPM) was rarely reported in patients with HCC because of the poor prognosis of HCC. However, with advances in early screening and therapeutic options for HCC, EHPM is increasingly being diagnosed in the clinic during the initial diagnosis of HCC or after curative treatment. Recent studies revealed that the incidence of EHPM in patients with HCC has increased in many countries in recent decades [2-12], and therefore, proper screening and treatment strategies for EHPM in patients with HCC represent an issue worthy of increased attention.

Warren and Gates defined the criteria for multiple primary malignant neoplasia as follows: 1) each tumor must definitively exhibit malignancy; 2) each tumor must be distinct; and 3) the probability of a tumor being a secondary metastatic lesion of the other tumor must be reasonably excluded [13]. Therefore, EHPM tumors must arise outside the liver, they must be clearly identifiable at the site of origin, and they must be correctly diagnosed histologically. Two previous studies including subjects diagnosed between 1980 and the mid-1990s in North America and Japan reported the prevalence (5.5–8.9%) of EHPM in patients with HCC and diverse clinical features [7,12]. Interestingly, the overall survival of patients with HCC was not altered by the presence of EHPM in either study, and death was more commonly related to HCC rather than EHPM, suggesting the extremely poor prognosis of HCC negated the prognosis of EHPM in most patients.

The epidemiology and etiology of HCC differ among countries, and the prognosis of HCC is improving. Moreover, a few studies of EHPM in patients with HCC in the Asia-Pacific regions excluding Japan and Taiwan have been reported [2,4,7,8,10,11,14-16]. This study aimed to elucidate the prevalence, clinical characteristics, and outcomes of EHPM in patients with surgically resected HCC in Korea, where the major cause of HCC is hepatitis B virus (HBV) infection [17].

## Methods

### Patients

In total, 270 patients with pathologically proven HCC underwent surgical resection in Seoul National University Bundang Hospital between May 2003 and December 2011. Among them, 20 patients were excluded; 10 patients underwent liver transplantation, 2 patients underwent palliative surgery, and 8 patients displayed combined HCC and cholangiocarcinoma. Therefore, the final study population included 250 patients who underwent curative surgical resection for HCC.

### Methods

The clinical characteristics of the patients, presence of EHPM, overall survival, and cause of death were retrospectively analyzed. Patient demographics, HCC etiology, and biochemical laboratory data were retrieved from electronic medical records. The etiology of HCC was classified as HBV or hepatitis C virus (HCV) on the basis of the serological presence of hepatitis B surface antigen or anti-HCV antibody, respectively, or alcohol on the basis of a history of alcohol intake of more than 80 g/day for men and 40 g/day for women for more than 10 years [18]. Survival and mortality, including the cause of death, were confirmed by an examination of the final medical records or via telephone calls to the participants or their family members. Overall survival was defined as the interval between the date of HCC surgery and the date of death or the last follow-up. The mean follow-up duration was 46.8 months (range 0–119 months). This study was approved by the institutional review board of Seoul National University Hospital.

We diagnosed EHPM according to the criteria given by Warren and Gates [13]. The EHPM group was further subdivided to prior, synchronous, and metachronous groups by using a 6-month interval between the diagnoses of the primary and secondary cancers [7,12]. Prior EHPM was defined as EHPM diagnosed more than 6 months prior to the diagnosis of HCC, synchronous EHPM was defined as EHPM diagnosed within 6 months before or after the diagnosis of HCC, and metachronous EHPM was defined as EHPM diagnosed more than 6 months after the diagnosis of HCC. Prior EHPM was identified by evaluating the patients’ medical records or history, and synchronous and metachronous EHPM were detected by preoperative or postoperative screening of radiological images and histological confirmation.

### Statistical analysis

Descriptive data were presented as the mean ± standard deviation or number (percentage). The chi-squared test and Student *t*-test were applied to analyze parametric data, and the Fisher exact test and Mann–Whitney U test were used for nonparametric data. The Kaplan-Meier method and Cox regression analysis were applied for survival analyses. All statistical results were analyzed by using SPSS version 20.0 (SPSS Inc., Chicago, IL, USA).

## Results

### Prevalence and clinical characteristics of EHPM in patients with HCC

In total, 33 of 250 patients (13.2%) with surgically resected HCC presented with EHPM. Of these, 32 patients had a single EHPM, and the remaining patient had 2 EHPMs (stomach and colon cancers). We divided the subjects according to the presence (n = 33) and absence of EHPM (n = 217), and compared the clinical and pathologic features of the two groups. Compared to the non-EHPM group, the EHPM group was significantly older, and patients in this group had more comorbidity such as diabetes mellitus and hypertension, a lower proportion of HBV etiology, lower serum levels of alanine aminotransferase, total bilirubin, and aspartate aminotransferase (AST), and a lower AST to platelet ratio index. However, tumor size, the frequency of vascular invasion, and the pathologic TNM stage of HCC were not significantly different between the 2 groups (Table 1).

**Table 1:** **Clinical characteristics of 250 surgically resected HCC patients according to the existence of EHPM**

Variables	non-EHPM group (n = 217)	EHPM group (n = 33)	*p*-value
Age, years^a, †^	55.1 ± 11.1	63.1 ± 10.6	<0.001^*^
Male sex^b^	168 (77.4)	25 (75.8)	0.832
Diabetes mellitus^b^	36 (16.6)	12 (36.4)	0.007^*^
Hypertension^b^	60 (27.6)	16 (48.5)	0.015^*^
Coexisting cirrhosis^b^	126 (58.1)	21 (63.6)	0.545
Etiology of liver disease^b^			<0.001^*^
HBsAg (+)	167 (77.0)	16( 48.5)	
Anti-HCV (+)	19 (8.8)	2 (6.1)	
Alcohol	9 (4.1)	6 (18.8)	
Other	22 (10.1)	9 (27.30)	
CTP score^b^			1.000
A	206 (94.9)	32 (97.0)	
B	11 (5.1)	1 (3.0)	
MELD score^a^	6.1 ± 3.4	5.4 ± 3.3	0.210
AST (IU/L)^a^	54.9 ± 74.1	33.6 ± 13.8	0.024^*^
ALT (IU/L)^a^	55.8 ± 90.1	31.3 ± 17.4	0.016^*^
Total bilirubin (mg/dL)^a^	1.5 ± 7.1	0.7 ± 0.3	0.020^*^
Serum albumin (g/dL)^a^	4.0 ± 0.5	3.9 ± 0.5	0.126
Platelet count (×10^3^/μL)^a^	162.7 ± 72.7	171.2 ± 65.6	0.439
APRI score^a^	1.1 ± 1.4	0.6 ± 0.3	0.034^*^
AFP (ng/mL)^b^			0.149
<20	109 (50.9)	20 (64.5)	
20–200	41 (19.2)	5 (16.1)	
>200^b^	64 (29.9)	6 (19.4)	
pTNM stage of HCC^b^			0.259
I	106 (40.8)	19 (57.6)	
II	77 (35.5)	11 (33.3)	
III or IV	34 (15.7)	3(9.1)	
Portal vein or major vessel invasion^b^	11 (5.1)	3 (9.1)	0.406
Microvessel invasion^b^	86 (39.6)	12 (36.4)	0.720
Tumor size^a^	4.2 ± 2.8	3.8 ± 2.5	0.477
Follow-up duration (months)^a^	48.5 ± 26.2	36.0 ± 30.0	0.013^*^
Overall median survival (months)	46.0	27.0	0.178
HCC recurrence^b^	114 (52.5)	10 (30.3)	0.017^*^
Time to recurrence (months)^a^	17.8 ± 16.8	21.4 ± 17.5	0.548
Number of deaths^b^	54 (24.9)	11 (33.3)	0.303
Time to death (months)^a^	29.8 ± 22.6	15.2 ± 15.3	0.045^*^
Cause of death^b^			0.005^*^
Liver-related	50 (92.6)	6 (54.5)	
Other	4 (7.4)	5 (45.5)	

*AFP*, alpha-fetoprotein; *ALT*, alanine aminotransferase; *APRI*, AST to platelet ratio index; *AST*, aspartate aminotransferase; *CTP*, Child-Turcotte-Pugh; *EHPM*, extrahepatic primary malignancy; *HBsAg*, hepatitis B surface antigen; *HCC,* hepatocellular carcinoma; *MELD*, model for end-stage liver disease; *pTNM stage*, pathologic TNM stage.

^a^mean ± standard deviation.

^b^percent.

^†^At the time of HCC diagnosis.

^*^p < 0.05.

**Figure 1:**
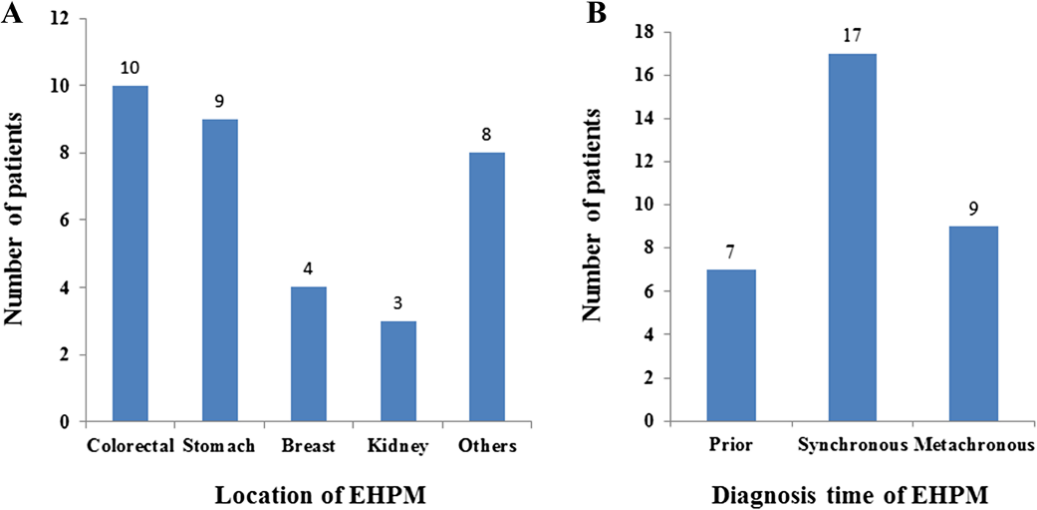
**Classification of 33 EHPM patients according to the location and diagnosis time of EHPM. (A)** The most common site of extrahepatic primary malignancy (EHPM) in patients with HCC was the colorectal (30.3%), followed by the stomach (27.3%), breasts (12.1%), and kidneys (9.1%). **(B)** Prior, synchronous, and metachronous EHPM were found in 7, 17, and 9 patients, respectively. Prior EHPM was defined as EHPM diagnosed more than 6 months prior to the diagnosis of HCC, synchronous EHPM was defined as EHPM diagnosed within 6 months before or after the diagnosis of HCC, and metachronous EHPM was defined as EHPM diagnosed more than 6 months after the diagnosis of HCC.

The most common site of EHPM in patients with HCC was the colorectal (30.3%), followed by the stomach (27.3%), breasts (12.1%), and kidneys (9.1%). Prior, synchronous, and metachronous EHPM were found in 7, 17, and 9 patients, respectively (Figure 1). The detailed tumor location, characteristics, pathologic TNM stage and applied treatment modality for EHPM are described in Tables 2 and 3. Most patients with EHPM underwent curative surgery (84.8%) and one third of EHPM group (33.4%) showed advanced tumor stage (≥TNM stage 3).

**Table 2:** **Clinical Characteristics and outcome of the HCC patients with EHMP**

Variables	Prior^†^EHPM group (n = 7)	Synchronous^†^EHPM group (n = 17)	Metachronous^†^EHPM group (n = 9)	Total (n = 33)	*p*-value
Age, years^a, ‡^	64.3 ± 2.6	62.5 ± 2.8	63.3 ± 4.2	63.1 ± 1.8	0.936
Male sex^b^	4 (57.1)	14 (82.4)	7 (77.8)	25 (75.8)	0.487
Smoking, >10 pack-years^b^	3 (42.9)	6 (35.3)	3 (37.5)	12 (37.5)	1.000
Etiology of liver disease^b^					0.471
HBsAg (+)	5 (71.4)	6 (35.3)	5 (55.6)	16 (48.5)	
Anti-HCV (+)	1 (14.3)	1(5.9)	0 (0.0)	2 (6.1)	
Alcohol	0 (0.0)	5(29.4)	1 (11.1)	6 (18.2)	
Other	1 (14.3)	5(29.4)	3 (33.3)	9 (27.3)	
CTP score^b^					0.212
A	6 (85.7)	17 (100.0)	9 (100.0)	32 (97.0)	
B	1 (14.3)	0 (0.0)	0 (0.0)	1 (3.0)	
pTNM stage of HCC^b^					0.191
I	2 (28.6)	12 (70.6)	5 (55.6)	19 (57.6)	
II	4 (57.1)	3 (17.6)	4 (44.4)	11 (33.3)	
III or IV	1 (14.3)	2 (11.8)	0 (0.0)	3 (9.1)	
AFP (ng/mL)^b^					0.470
<20	5 (71.4)	11 (68.8)	4 (50.0)	20 (64.5)	
20–200	0 (0.0)	2 (12.5)	3 (37.5)	5 (16.1)	
>200	2 (28.6)	3 (18.8)	1 (12.5)	6 (19.4)	
Location of EHPM^b^					
Esophagus	0 (0.0)	1 (5.9)	0 (0.0)	1 (3.0)	1.000
Stomach	4 (57.1)	4 (23.5)	1 (11.1)	9 (27.3)	0.152
Colorectal	1 (14.3)	6 (35.3)	3 (33.3)	10 (30.3)	0.697
Pancreas	0 (0.0)	0 (0.0)	1 (11.1)	1 (3.0)	0.485
Common bile duct	0 (0.0)	0 (0.0)	1 (11.1)	1 (3.0)	0.485
Breast	2 (28.6)	2 (11.8)	0 (0.0)	4 (12.1)	0.222
Thyroid	0 (0.0)	0 (0.0)	1 (11.1)	1 (3.0)	0.485
Kidney	1 (14.3)	2 (11.8)	0 (0.0)	3 (9.1)	0.579
Bladder	0 (0.0)	0 (0.0)	1 (11.1)	1 (3.0)	0.485
Prostate	0 (0.0)	0 (0.0)	1 (11.1)	1 (3.0)	0.485
Retroperitoneum	0 (0.0)	1 (5.9)	0 (0.0)	1 (3.0)	1.000
Bone marrow	0 (0.0)	1 (5.9)	0 (0.0)	1 (3.0)	1.000
pTNM stage of EHPM					0.166
0	0 (0.0)	1 (5.9)	0 (0.0)	1 (3.3)	
1	4 (80.0)	8 (47.1)	1 (12.5)	13 (43.3)	
2	1 (20.0)	4 (23.5)	1 (12.5)	6 (20.0)	
3	0 (0.0)	3 (17.6)	5 (62.5)	8 (26.7)	
4	0 (0.0)	1 (5.9)	1 (12.5)	2 (6.7)	
Treatment of EHPM^b^					1.000
Operation	7 (100.0)	14 (82.4)	7 (77.8)	28 (84.8)	
Radiation or Chemotherapy	0 (0.0)	1 (5.9)	1 (11.1)	2 (6.1)	
Supportive care	0 (0.0)	2 (11.8)	1 (11.1)	3 (9.1)	
Progression or recurrence of EHPM^b^	0 (0.0)	6 (35.3)	3 (33.3)	9 (27.3)	0.221
Follow-up duration (months)^a^	18.4 ± 5.9	35.9 ± 6.7	49.8 ± 12.5	36.0 ± 5.2	0.115
Overall median survival (months)	18.0	32.0	38.0	28.0	
Recurrence of HCC^b^	3 (42.9)	5 (29.4)	2 (22.2)	10 (30.3)	0.781
Death^b^	2 (28.6)	7 (41.2)	2 (22.2)	11 (33.3)	0.628
Cause of death^b^					0.697
Liver-related	1 (50.0)	3 (42.9)	2 (100.0)	6 (100.0)	
Other	1 (50.0)	4 (57.1)	0 (0.0)	5 (100.0)	

*AFP*, alpha-fetoprotein; *CTP*, Child-Turcotte-Pugh; *EHPM*, extrahepatic primary malignant neoplasm; *HBsAg*, hepatitis B surface antigen; *HCC*, hepatocellular carcinoma; *pTNM stage*, pathologic TNM stage.

^a^mean ± standard deviation, ^b^percent.

^†^Prior EHPM group, EHPM developed more than 6 months before the diagnosis of HCC; Synchronous EHPM group, EHPM developed within 6 months of the diagnosis of HCC; Metachronous EHPM group, EHPM developed more than 6 months after the diagnosis of HCC.

^‡^At the time of HCC diagnosis.

^*^p < 0.05.

**Table 3:** **Locations and pathologic TNM stages of 33 EHPM patients**

Case number	EHPM site	EHPM group^a^	pTNM stage^†^				Follow up or survival duration (months)	Cause of death^b^
			T	N	M	Stage		
1	Rectum	S	3	0	0	IIA	118	
2^*^	Breast	S	1	0	0	IA	4	H
3^*^	Stomach	S	3	3	1	IV	10	E
4^*^	Stomach	S	1	0	0	IA	21	E and O
5	Breast	S	1	0	0	IA	53	
6^*^	Kidney	S	1	0	0	I	0	H
7^*^	Rectum	S	3	2	0	IIIB	12	H
8	Esophagus	S	1	0	0	IA	52	
9	Kidney	S	1	0	0	I	51	
10	Colon	S	3	0	0	IIA	48	
11	Stomach (MALToma)	S				IEA(Ann Arbor)	47	
12	Colon	S	3	0	0	IIA	32	
13	Colon	S	3	0	0	IIA	28	
14	Retroperitoneum	S	2b	0	0	Ib	35	
15^*^	Multiple myeloma	S				III(ISS)	23	E
16	Stomach	S	is	0	0	0	17	
17^*^	Rectum	S	3	1	0	IIIB	4	O
18^*^	Rectum	S	4b	2b	0	IIIC	3	H and E
19^*^	Stomach	M	3	0	0	IIA	16	H
20^*^	Kidney	P	NA				18	H
21	Pancreas	M	3	1	0	IIIB	55	
22	Prostate	M	3	0	0	III	74	
23	Breast	P	2	0	0	IIA	46	
24	Colon	P	2	0	0	I	27	
	Stomach	P	1b	0	0	IA		
25	Colon	M	3	0	1	IVA	38	
26	Colon	M	NA				24	
27	Thyroid	M	3	1a	0	III	29	
28	CBD	M	4	1	0	III	109	
29	Stomach	P	1	0	0	IA	17	
30	Stomach	P	NA				0	
31^*^	Breast	P	1	0	0	IA	1	O
32	Stomach	P	1	0	0	IA	20	
33	Bladder	M.	1	0	0	I	100	

*CBD,* common bile duct; *EHPM*, extrahepatic primary malignant neoplasm; *HCC*, hepatocellular carcinoma; *is*, in situ; *NA,* not applicable; *pTNM stage*, pathologic TNM stage.

^a^M, metachronous group; P, prior group; S, synchronous group.

^b^H, HCC related; E, EHPM related; O, Others.

^†^Based on AJCC 7th edition, except Ann Arbor Staging for gastric lymphoma (MALToma) and ISS (International Staging System) for multiple myeloma.

*Patient who expired.

All patients in the prior EHPM group underwent curative resection and did not display any recurrence of the malignancy. Although not statistically significant, mortality was highest in the synchronous EHPM group in which extrahepatic causes of death were more frequently observed.

### Clinical outcomes of patients with HCC and EHPM

**Figure 2:**
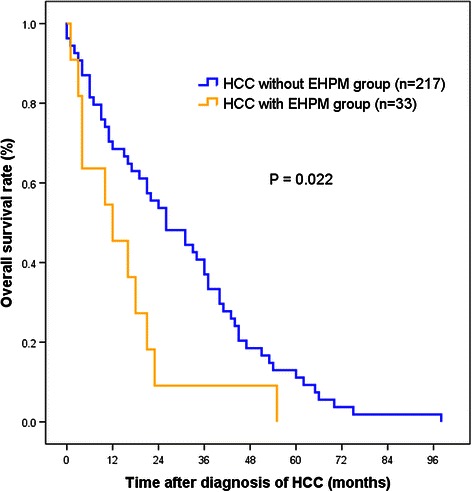
**Comparison of the overall survival between the HCC groups with and without EHPM.** Using Kaplan Meier analysis, the cumulative probabilities of overall survival at 1 year, 3 years, and 5 years in the EHPM group were 45.5%, 9.1%, and 0%, respectively, compared to 79.6%, 37.0%, and 11.1%, respectively, in the non-EHPM group. EHPM group showed poorer survival compared to non-EHPM group.

In total, 65 patients (26%) died during the mean follow-up period of 46.8 months. The median overall survival was 27 months in the EHPM group versus 46 months in the non-EHPM group (*p* = 0.178). The cumulative probabilities of overall survival at 1 year, 3 years, and 5 years in the EHPM group were 45.5%, 9.1%, and 0%, respectively, compared to 79.6%, 37.0%, and 11.1%, respectively, in the non-EHPM group (Figure 2). To confirm the role of EHPM in overall survival, univariate and multivariate analyses of mortality were performed. The presence of EHPM was an independent factor for mortality in multivariate analysis in addition to microvessel invasion and alpha-fetoprotein levels exceeding 20 ng/mL (Table 4). The recurrence rate of HCC was significantly higher in the non-EHPM group. The cause of death was distinct between the 2 groups. Almost all patients in the non-EHPM group died of liver-related problems, whereas approximately half (5 of 11) patients in the EHPM group died of non-liver–related causes, including EHPM progression (n = 2, 18%), brain hemorrhage (n = 1, 9%), sepsis (n = 1, 9%), and sudden cardiac arrest (n = 1, 9%).

**Table 4:** **Factors associated with mortality among 250 patients with HCC (surgically resected between May 2003 and Dec 2011)**

Variables	Univariate analysis^†^				Multivariate analysis^†^	
	HR	95% CI	p value	HR	95% CI	*p*-value
Age > 60 years	0.967	0.578–1.616	0.897			
Male sex	0.777	0.394–1.533	0.468			
Diabetes mellitus	1.602	0.880–2.916	0.123			
Hypertension	1.172	0.699–1.965	0.547			
Alcohol drinking	0.676	0.405–1.128	0.134			
Smoking, >10 pack-years	1.069	0.643–1.777	0.796			
Portal vein or major vessel invasion	3.073	1.317–7.172	0.009^*^	1.066	0.361–3.151	0.908
Microvessel invasion	1.983	1.188–3.310	0.009^*^	1.850	1.058–3.236	0.031^*^
Intrahepatic metastasis	1.609	0.923–2.804	0.094			
Alpha-fetoprotein > 20 ng/mL	1.806	1.083–3.014	0.043^*^	1.716	1.018–2.892	0.043^*^
Child-Turcotte-Pugh score B or C	0.840	0.361–1.955	0.686			
Presence of EHPM	2.125	1.087–4.155	0.028^*^	2.002	1.016–3.942	0.045^*^
pTNM stage > II	2.303	1.285–4.129	0.005^*^	1.784	0.950–3.351	0.072

*CI*, confidence interval; *EHPM*, extrahepatic primary malignant neoplasm; *HCC*, hepatocellular carcinoma; *HR*, hazard ratio; *pTNM* score, pathologic TNM stage.

^†^According to the Cox proportional hazard model.

^*^p < 0.05.

## Discussion

In this study, we demonstrated that the prevalence of EHPM in patients who underwent curative resection for HCC in Korea was 13.2%, which was higher than that reported previously. The most common locations of EHPM were the colorectal, stomach, breasts, and kidneys. The overall survival of the EHPM group was significantly worse than that of the non-EHPM group, and the cause of death was non-liver–related in approximately half of the patients in the EHPM group. Moreover, EHPM was an independent factor for overall survival in multivariate analysis.

Our study applied the criteria of Warren and Gates to identify EHPM [13]. By using the same criteria, a North American retrospective study found that 74 of 1349 (5.5%) patients with HCC also presented with EHPM between 1980 and 1993. The patients with EHPM tended to be older and of the male sex, and the most common EHPMs were prostate cancer (n = 16) and colorectal cancer (n = 15). However, no significant difference in mean survival according to the presence of EHPM was noted [12]. A study in Japan also used the same criteria and identified 41 EHPMs in 463 (8.9%) patients with surgically resected HCC between 1979 and 1994. No significant differences in clinical variables and survival were noted between the EHPM and non-EHPM groups. In this study, the most common EHPMs were gastric (n = 18) and colorectal (n = 9) cancers [7]. On the other hand, the International Agency for Research on Cancer (IARC) criteria for a second primary neoplasm are as follows: 1) the existence of two or more primary cancers does not depend on time; 2) a primary cancer is a cancer that originates in a primary site or tissue and is not an extension, recurrence, or metastasis of another cancer; 3) only one tumor shall be recognized as arising in an organ or a pair of organs or tissues, which does not apply if the tumors in an organ are of different histologies; and 4) the second neoplasm must have a different histological type than the primary lesion in the pathological diagnosis [19]. These rules have been adapted in studies with large registry data, including a retrospective cohort study in China and a nationwide cohort study in Taiwan, which may include non-histologically proven tumors [2,3]. According to either the IARC or Warren and Gates criteria, the prevalence of EHPM in patients with HCC was 1.6–25.7% in previous studies [2-12,14-16,20-23]. In our study, the prevalence of EHPM was 13.2%, which was noticeably higher than those reported in other Asian countries, in which the prevalence is typically less than 10% [2-4,7,8,10,11,14-16]. Although the reason for this higher prevalence is unclear, as many of the previous studies were performed 20 years earlier than our study, this difference could be related to the increasing number of cancer survivors as a result of advances in early detection and various treatment options for cancer.

Concerning the most common locations of EHPM in patients with HCC, colorectal cancer was the most prevalent EHPM in this study, followed by gastric and breast cancers [2]. In a study conducted in Spain, colorectal cancer was the most prevalent EHPM in patients with HCC followed by head and neck cancer and genitourinary cancer, in line with the distribution of cancer in the general population of Spain [5]. A report from South China unexpectedly identified nasopharyngeal cancer as the most prevalent EHPM in patients with HCC, although the incidence of nasopharyngeal cancer was high in the region, emphasizing the need for head and neck cancer screening among HCC survivors [3]. On the contrary, Di Stasi et al. reported 10 immunoproliferative cancers of B-cell origin among 35 EHPMs [21]. It is commonly suggested that the location of EHPM is similar to that of the general population; in particular, the Asian population has a high prevalence of gastric adenocarcinoma, whereas genitourinary and colorectal cancers are most prevalent EHPMs in Western countries [2,5,12]. Our data revealed colorectal cancer (30.3%) as the most popular EHPM in patients with surgically resected HCC followed by stomach and breast cancers (21.2% and 12.1%, respectively), suggesting that the location of EHPM in HCC also follows the typical distribution of cancers in the general population [24]. Therefore, gastric and colon cancer screening should be considered for patients with newly diagnosed HCC.

In our study, the EHPM group was older, and these patients presented with more accompanying comorbidities, various etiologies of liver disease, preserved liver function, and lower recurrences of HCC but higher mortality rates compared to the non-EHPM group. Some studies reported a higher mean age for the EHPM group [4,10,12], but this finding was not consistent [5,11]. Recent reviews emphasized age as a risk factor for the development of multiple primary malignant neoplasms [25,26]. Accompanying chronic diseases such as diabetes mellitus [7] or chronic kidney disease [2] have been reported in other studies, and in this study, diabetes mellitus and hypertension were more common in the EHPM group. Moreover, Andrykowski reported poorer mental health status as well as an increased number of lifetime comorbidities in patients with multiple primary cancers compared to patients with a single cancer or no cancer [27]. Therefore, integrated care including the treatment of mental health problems and comorbidities is required for patients with HCC and EHPM.

Although HBV was the predominant etiology in the non-EHPM group (63.6%), the etiology differed greatly in the EHPM group, which had a greater proportion of cancers related to alcohol (18.8%) or of unknown etiology (24.2%) and fewer cancers associated with HBV (33.3%). This difference may be related to the older age of patients in the EHPM group. This finding is comparable with those of other studies [8], but inconsistent reports also exist [5,7]. Some studies reported a higher prevalence of liver cirrhosis in the EHPM group [8,12], although this finding is controversial [4,8,10].

One of the most peculiar findings of this study was the significantly poorer survival of the EHPM group compared to that of the non-EHPM group. To our knowledge, no previous study reported poorer survival for patients with EHPM because the prognosis of HCC is generally worse than that of EHPM [3,5-7,12]. Some previous studies reported even higher overall survival for the EHPM group [5,6]. In our study, both the cumulative probability of survival and the result of multivariate analysis concomitantly support the poor survival of the EHPM group. We cannot figure out the exact cause of the poor outcome of EHPM group. However, several characteristics could be found in the eleven expired patients of EHPM group. First, all of these eleven patients lived no longer than two years. Second, their causes of death were much more related with EHPM progression or other reasons such as peritoneal seeding of stomach cancer, progression of multiple myeloma, sepsis after operation, sudden cardiac arrest or brain hemorrhage rather than liver related causes. Third, higher proportion of advanced stages (stage 3 or 4) of EHPM existed in these eleven patients. From these points, we can suppose that EHPM could have hazardous effect on survival of HCC patients and advanced stage of EHPM should be alarmed in survival of HCC patients. Therefore, we can suggest that surveillance for EHPM in HCC patients should be reinforced and early detection and treatment of EHPM possibly benefit the survival of HCC patients.

The limitations of this study were its retrospective design and relatively small number of enrolled patients, in addition to the inclusion of only patients with surgically resected HCC. We could not recruit the details of the screening pattern of EHPM of each patient. However, this study is the first report of the clinical and pathological features and outcomes of patients with HCC and EHPM in Korea, where HBV is the most common cause of liver disease, in addition to the country’s rapid socioeconomic development and improvements in the diagnosis and treatment of HCC.

## Conclusions

In conclusion, the prevalence of EHPM in patients with HCC who underwent curative resection for HCC in Korea was 13.2%, which was higher compared to previous reports. The overall survival of patients with HCC and EHPM was significantly worse than that of patients with HCC without EHPM. Therefore, considering the high prevalence of EHPM and its adverse effect on overall survival, proper screening strategy for early detection and treatment of EHPM should be emphasized in patients with HCC.
